# Trends and Prospects in Inflammation Resolution in Acute Lung Injury and Acute Respiratory Distress Syndrome: A Bibliometric Quantitative and Visualization Analysis

**DOI:** 10.1155/mi/2918784

**Published:** 2026-04-02

**Authors:** Zixin Luo, Nan Huang, Kaihang Luo, Chenxi Wang, Jianhua Li, Kang Zou

**Affiliations:** ^1^ The First Clinical Medical College, Gannan Medical University, Ganzhou, 341000, Jiangxi, China, gmu.cn; ^2^ Department of Critical Care Medicine, Medical Center of Anesthesiology and Pain, Jiangxi Medical College, The First Affiliated Hospital, Nanchang University, Nanchang, 330000, Jiangxi, China, ncu.edu.cn; ^3^ Jiangxi Provincial Key Laboratory of Critical Care Medicine for Health and Wellness, Jiangxi Medical College, The First Affiliated Hospital, Nanchang University, Nanchang, 330000, Jiangxi, China, ncu.edu.cn; ^4^ Department of Critical Care Medicine, The First Affiliated Hospital of Gannan Medical University, Ganzhou, 341000, Jiangxi, China, gmu.cn

**Keywords:** ALI/ARDS, bibliometric, CiteSpace, drug delivery systems, inflammatory resolution, VOSviewer

## Abstract

**Background:**

Acute lung injury and acute respiratory distress syndrome (ALI/ARDS) are common and critical pulmonary conditions involving numerous inflammatory factors and oxidative stress responses. Inflammatory responses and oxidative stress are closely related to the development of ALI/ARDS and form an important theoretical basis for treatment and drug development. Although there has been extensive research on the resolution of inflammation in ALI/ARDS, no systematic bibliometric analysis has been conducted in this field.

**Methods:**

The researchers used bibliometrics to search the Web of Science Core Collection (WOSCC) database for research literature on the resolution of inflammation in ALI/ARDS from 2005 to 2024. Visualization mapping analysis was performed using tools such as CiteSpace and VOSviewer to analyze authors, research institutions, countries, journals, cocited literature, and keywords.

**Results:**

A total of 375 articles were included. The research showed an upward trend, with the highest number of publications in 2022. The United States took the leading position in this field, followed by China. JIN SW had the highest number of publications, while D’Alessio FR had the highest citation count. Harvard University had the highest intermediary centrality, the American Journal of Physiology‐Lung Cellular and Molecular Physiology published the most articles, and the American Journal of Respiratory and Critical Care Medicine had the highest impact factor. Resolvin D1 (RvD1) and Resolvin E1 (RvE1) played a key role in the resolution of inflammation. Drug delivery systems (DDSs), such as black phosphorus nanosheets (BPNSs) and liposomes, could efficiently deliver these mediators to enhance therapeutic effects and reduce side effects.

**Conclusion:**

Over the past 20 years, interest in the resolution of inflammation in ALI/ARDS has grown. The United States has dominated research in this area. The study of RvD1 and RvE1 has become a hot topic, and the development of DDSs has provided new strategies for clinical treatment.

## 1. Introduction

Acute lung injury and acute respiratory distress syndrome (ALI/ARDS) are prevalent and critical pulmonary conditions characterized by acute inflammatory responses [1]. In ALI/ARDS, pathological changes occurring in the lungs include extensive alveolar injury, which involves damage to type I alveolar epithelial cells and pulmonary vascular endothelial cells, interstitial fibrosis, and proliferation of type II epithelial cells, which give rise to edema and inflammatory cell infiltration of the lung tissue [2]. Recovery from ALI involves regressive and reparative phases, which may include severe pulmonary fibrosis [3]. There are no specific drugs for ARDS, and its inflammation is mainly dependent on the patient’s own defense mechanism, which is a spontaneous recovery process [4]. Endogenous lipid autocrine mediators, called arachidonoids, play a key role in the induction of inflammation and proinflammatory cytokine production [5, 6]. As new endogenous specialized proresolving lipid mediators (SPMs) are identified, such as arachidonic acid‐derived lipoxins (LXs) and omega‐3 fatty acid‐derived resolvins (Rvs), protectins (PDs), maresins (MaRs), CO, HO‐1, and ANXA1, we have been provided with a new perspective to understand the process of inflammation resolution [7, 8]. For example, Resolvin E1 (RvE1) promotes neutrophil apoptosis and accelerates the resolution of lung inflammation [9, 10]. These lipid mediators play a multifaceted role, not only regulating the infiltration and activation of inflammatory cells but also facilitating tissue repair and the clearance of alveolar fluid, which are essential for postlung injury recovery. For instance, lipid mediators such as Lipoxin A4 (LXA4) facilitate the resolution of lung injury by activating aquaporins and ion channels in alveolar epithelial cells, which enhance the clearance of alveolar fluid and thus aid in the repair process [11]. Therefore, investigating the advancements in research related to the resolution of inflammation in acute lung injury holds significant clinical implications for improving patient outcomes and reducing mortality rates.

In recent years, bibliometrics has gained increasing attention as a tool for analyzing trends and academic influence within the literature. A systematic analysis of relevant publications can reveal research hotspots, trends in evolution, and prospective avenues in a scholarly field [12, 13]. Recent bibliometric studies have spanned a range of conditions, including pulmonary diseases, cardiovascular disorders, and cancer [14–19]. However, there is a lack of bibliometric studies focusing on the resolution of inflammation in ALI/ARDS. This article aims to provide a comprehensive review of the research advancements in the resolution of inflammation in acute lung injury from a bibliometric perspective, with the hope of offering valuable information and insights to researchers and clinicians.

## 2. Materials and Methods

### 2.1. Data Sources and Search Strategies

**Figure 1:**
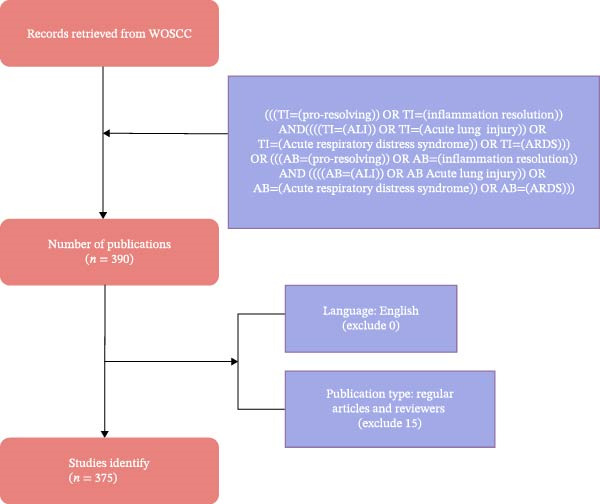
Diagram of the study selection process for inclusion criteria.

Leveraging the Web of Science Core Collection (WOSCC) database—renowned for its authoritative, comprehensive, and systematic nature in the academic community and widely utilized for visualizing scientific literature—we undertook a comprehensive search on the topic of “inflammation resolution and ALI/ARDS” on June 7, 2025. To ensure the credibility and accuracy of the research findings, we searched for articles with titles and abstracts using the following criteria: (((TI = (pro‐resolving)) OR TI = (inflammation resolution)) AND (((TI = (ALI)) OR TI = (Acute lung injury)) OR TI = (Acute respiratory distress syndrome)) OR TI = (ARDS)) OR (((AB = (pro‐resolving)) OR AB = (inflammation resolution)) AND (((AB = (ALI)) OR AB = (Acute lung injury)) OR AB = (Acute respiratory distress syndrome)) OR AB = (ARDS)), spanning from January 1, 2005, to December 31, 2024. Subsequently, we excluded non‐English articles and limited the publication types to reviews and original articles. Two researchers then independently conducted data searches and cleaning, discussed articles that were still contentious, and ultimately achieved a 90% consensus, demonstrating substantial agreement, resulting in a final selection of 375 articles (Figure 1).

### 2.2. Data Analysis

CiteSpace 6.3 R4 (developed by Chaomei Chen at Drexel University, Dalian, China), VOSviewer 1.6.16 (created by Van Eck and Waltman at the Centre for Science and Technology Studies, Netherlands), an online analytics platform (https://bibliometric.com/), R version 4.3.1, and Origin 2024 were utilized for data analysis and visualization.

CiteSpace primarily constructs co‐occurrence maps of institutions, dual‐map overlays of journals, cluster visualization graphs, timeline diagrams, and visualization maps of citation bursts. VOSviewer is mainly used for analyzing the co‐occurrence of authors, countries, journals, and keywords. R and https://bibliometric.com/ are primarily used for data analysis, calculating the H‐index and G‐index. Origin is used for plotting line graphs and bar charts of global publication numbers and average citation frequencies.

## 3. Results

### 3.1. Quantitative Analysis of Publications

**Figure 2:**
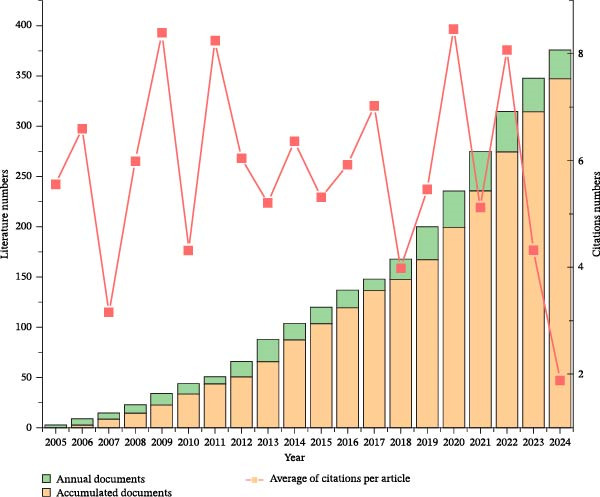
Count of papers, cumulative number of papers, and average citation frequency of articles per year from 2005 to 2024.

Over the 20‐year period from 2005 to 2024, a total of 375 articles were published on the topic of inflammation resolution in ALI/ARDS. Figure 2 illustrates the annual and cumulative number of publications related to this theme. Although there has been fluctuation in the annual publication count ranging from 2005 to 2024, the overall trend shows a steady increase, indicating a growing interest among researchers in the study of inflammation resolution in ALI/ARDS. Annual publication counts reached their zenith in 2022, registering 40 articles apiece, accompanied by average citation rates of 8.07 for each respective year.

### 3.2. Authors and Cociting Authors

The examination of the 375 selected publications disclosed that a total of 2571 authors have made contributions to this area of research. Table 1 lists the 10 authors with the highest publication output. JIN SW is the author with the most publications, as well as the highest H‐index and G‐index, while D’Alessio FR is the most cited author with a total citation count (TC) of 1441. Figure 3A presents a network of authors who have coauthored at least two publications, excluding those without collaborative ties, resulting in a final selection of 46 authors. Among them, Jin Sheng Wei has the strongest total link strength (98). After conducting a citation analysis with VOSviewer software, which included 68 authors with more than three publications, Figure 3B reveals that Jin Sheng Wei and Hao Y are the authors most relevant to the early research. Shang You has exerted significant influence during the mid‐period of the study, whereas Jin Sheng Wei has emerged as a recent author focusing on research related to the inflammation resolution in ALI/ARDS.

Figure 3Authors’ contributions to the study of inflammatory abatement in acute lung injury: (A) 46 coauthors who collaborated on more than two books and (B) 68 cociting authors with three or more publications.

**Figure:**
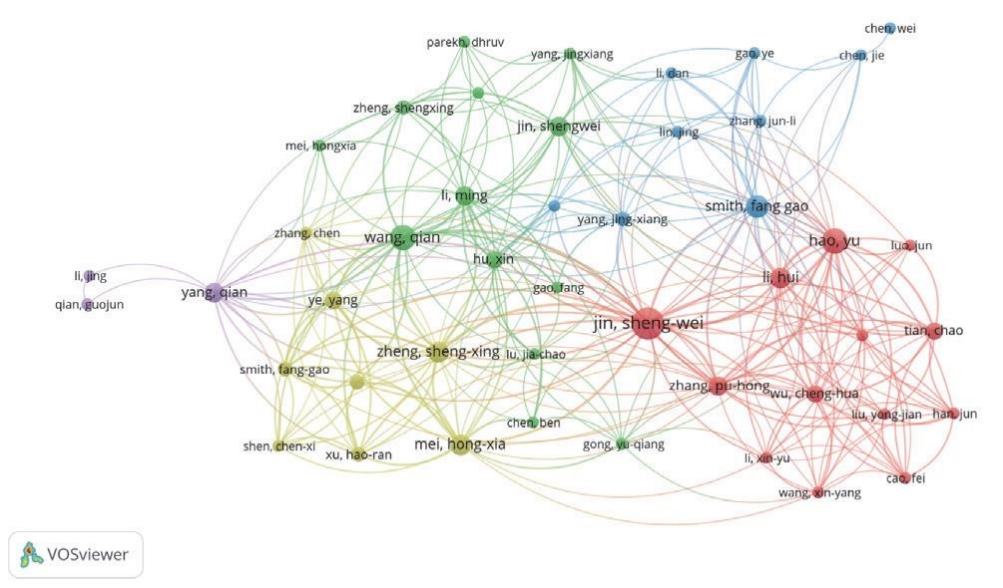
(A)

**Figure:**
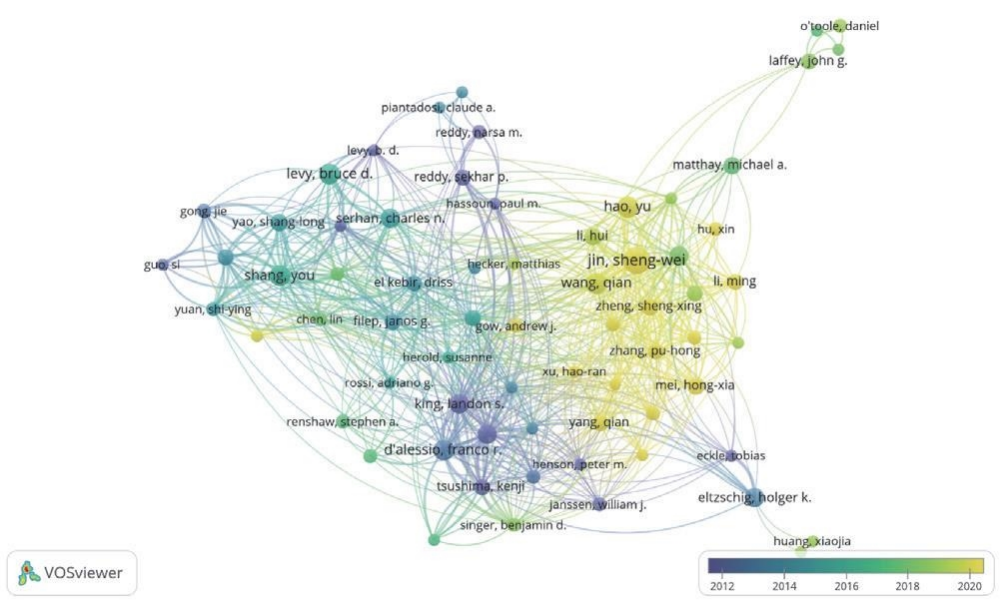
(B)

**Table 1:** Top 10 authors by number of publications.

Rank	Author	Publications	Tc	H‐index	G‐index	Country
1	Jin SW	20	489	15	20	China
2	Levy BD	12	1029	10	12	USA
3	Wang Q	11	265	9	11	China
4	Smith FG	10	253	9	10	England
5	Yao SL	10	224	7	10	China
6	D’Alessio FR	9	1441	9	9	USA
7	Hao Y	9	208	8	9	China
8	Zheng SX	9	225	8	9	China
9	Wang Y	9	171	7	9	China
10	Aggarwal NR	8	1422	8	8	USA

### 3.3. Analysis of Countries/Areas and Institutions

Table 2 highlights the 10 countries with the greatest number of publications on the topic of inflammation resolution in the context of ALI/ARDS, and the United States tops the list with the most publications in the field of inflammation resolution in ALI/ARDS (NC = 86), with China ranking second (NC = 85). Additionally, the United States leads in citation counts, H‐index, and G‐index, signifying a substantial impact of the United States in this area of research. In the study of inflammation resolution in ALI/ARDS, several countries have established international collaborations, as shown in Figure 4A. This chart depicts both the publication counts and the collaborative ties among nations, with the United States at the forefront for publication volume and exhibiting robust partnerships with China and other countries. Using VOSviewer software, with the number of publications set to one and excluding countries with no connections, Figure 4B presents a world map of the collaboration network among authors who have published papers together in 28 countries. Most of the countries on the map are from Europe. The size of the nodes reflects the number of publications, with larger nodes indicating more publications. The lines in the map represent collaborative relationships, with thicker lines indicating stronger collaborations. Figure 4C shows an institutional co‐occurrence map established by CiteSpace, with each node signifying a distinct institution. Within the CiteSpace visualization, node size corresponds to the frequency of institutional citations, while nodes with a purple ring indicate a higher level of betweenness centrality. Nodes with a betweenness centrality exceeding 0.1 are considered key nodes in CiteSpace due to their significant importance and influence [20, 21]. Table 3 summarizes the top 10 institutions by betweenness centrality, with Harvard University having the highest centrality score (centrality = 0.21). Among the top 10 institutions, six have a betweenness centrality greater than 0.1, and seven of them are from the United States.

Figure 4Collaboration between countries/regions and institutions. (A) String diagram for evaluating collaboration across countries. (B) World map of national collaboration networks. (C) Institutional co‐occurrence diagram created by CiteSpace.

**Figure:**
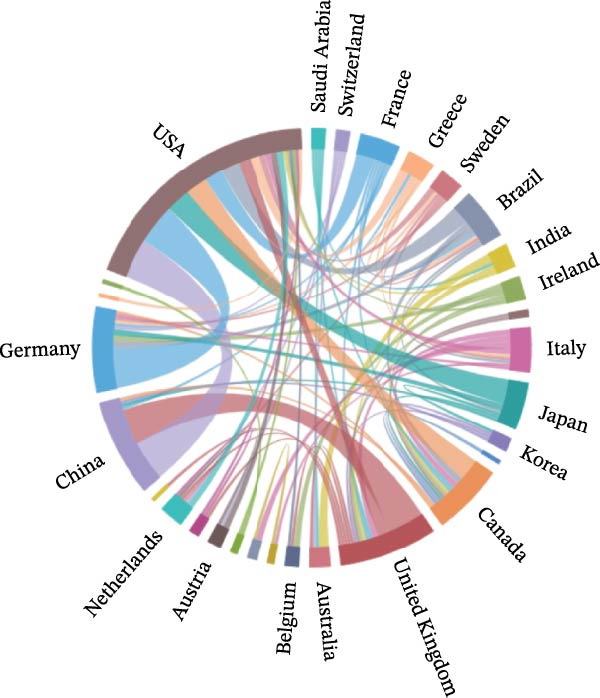
(A)

**Figure:**
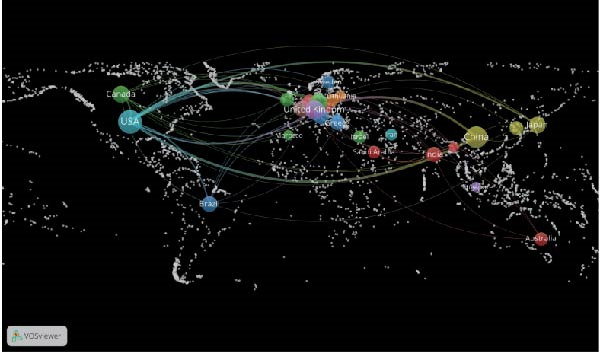
(B)

**Figure:**
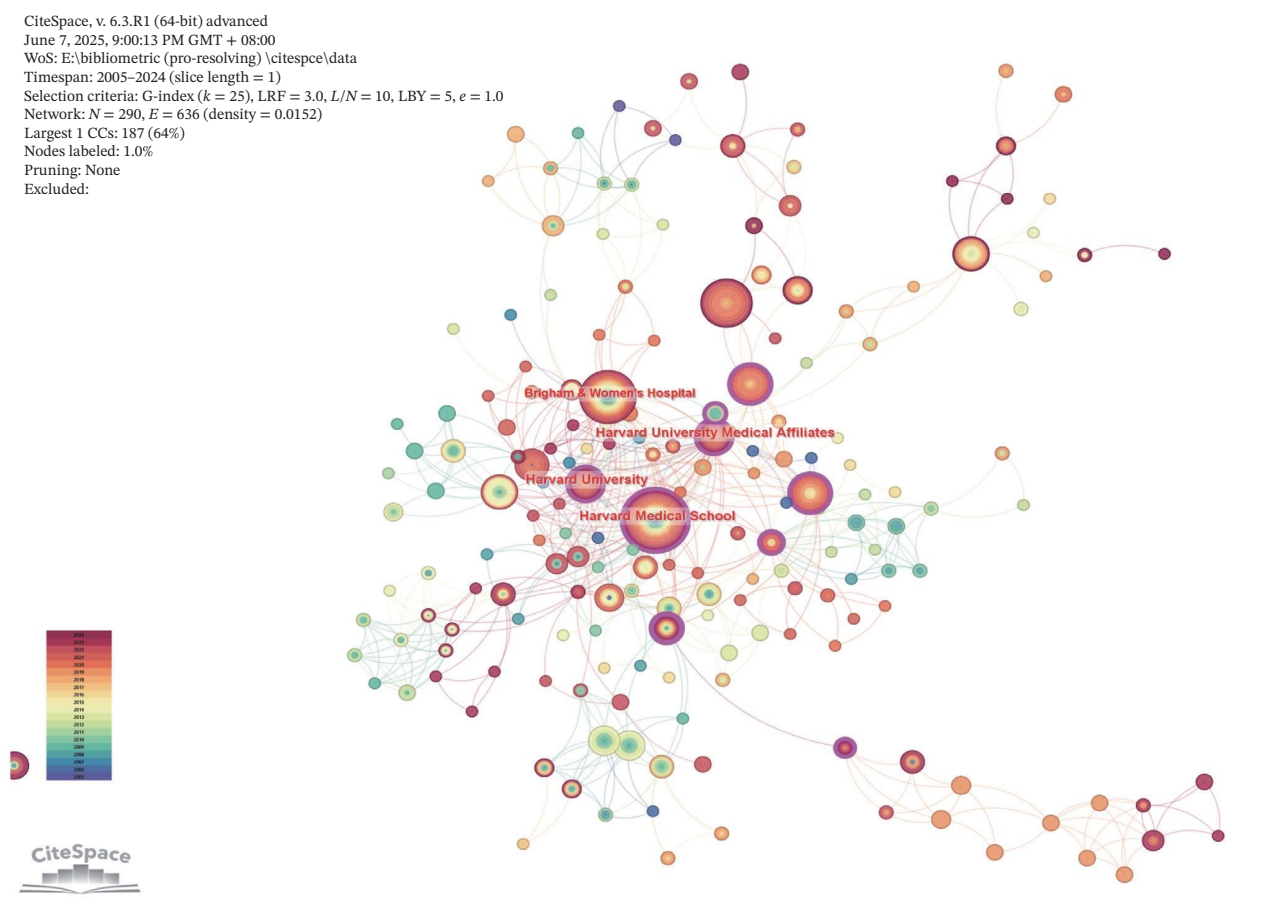
(C)

**Table 2:** Top 10 countries in terms of number of publications.

Rank	Country	No. of documents	Total citations	H‐index	G‐index
1	USA	86	3415	30	56
2	China	85	2015	26	40
3	France	12	875	11	12
4	Germany	11	277	8	11
5	United Kingdom	9	521	9	9
6	Brazil	8	126	5	8
7	Canada	8	259	5	8
8	India	7	77	6	7
9	Japan	6	131	5	6
10	Italy	5	382	5	5

**Table 3:** Top 10 affiliations in terms of intermediary centrality.

Rank	Affiliation	Freq	Degree	Centrality	Country
1	Harvard University	28	38	0.21	USA
2	University of Edinburgh	11	19	0.14	UK
3	Institut National de la Sante et de la Recherche Medicale (Inserm)	7	21	0.13	France
4	University of Southern California	3	7	0.13	USA
5	Baylor College of Medicine	3	5	0.11	USA
6	University of Birmingham	10	9	0.1	UK
7	Brigham & Women’s Hospital	23	33	0.09	USA
8	Huazhong University of Science and Technology	15	11	0.09	USA
9	Duke University	7	16	0.08	USA
10	University System of Ohio	9	15	0.08	USA

### 3.4. Analysis of Journals and Cocited Journals

Out of 187 journals, a total of 375 articles related to the resolution of inflammation and ALI/ARDS have been published. Table 4 presents the top 10 journals by the number of publications. The American Journal of Physiology‐Lung Cellular and Molecular Physiology leads in terms of publication volume, with a total of 16 articles, which also has the highest G‐index (G‐index = 16), followed by Frontiers in Immunology (*n* = 15). In addition, American Journal of Respiratory and Critical Care Medicine is the most frequently cited in terms of total citations (TC = 1304). The impact factor of a journal, denoted as IF, is a crucial metric for evaluating the influence of scholarly publications and is used to measure the reputation of a journal, to assess the quality of research, and to evaluate the impact of research results [22]. The American Journal of Respiratory and Critical Care Medicine topped the charts with an impact factor of 19.3 in 2024, followed by Proceedings of the National Academy of Sciences of the United States of America (IF 2024 = 9.4) and JCI Insight (IF 2024 = 6.3). Figure 5A analyzes journal cocitations through the VOSviewer software, including journals with publication volume greater than or equal to one (187 journals) and removing journals with no link between them, 129 journals were included in the analysis, of which Frontiers in Immunology had the strongest total link strength (total link strength = 52). Figure 5B presents a visualization of the cocitation network map for 183 journals with more than 20 citations, highlighting the collaborative research landscape in the field of inflammation resolution in ALI/ARDS. The three most frequently cited journals are the Journal of Immunology (1133), American Journal of Respiratory and Critical Care Medicine (891), and Journal of Clinical Investigation (596). The dual‐map overlay created by Chen and Leydesdorff facilitates the visualization of scientific pattern combinations at the discipline level for global scientific journal maps [23]. In the dual‐map overlay visualization focusing on inflammation resolution and ALI/ARDS, the citing journals are located on the left side, and the cited journals are on the right side. Labels within clusters represent the corresponding disciplines of the citing or cited journals, and the clusters themselves denote the respective disciplines. Connections between clusters represent the pathways of citation references, with the thickness of the lines being directly proportional to the frequency of citations [24, 25]. Figure 5C indicates that research published in journals within the Molecular/Biology/Genetics field is frequently cited by studies published in journals within the Molecular/Biology/Immunology and Medicine/Medical/Clinical fields.

Figure 5Journal contributions and collaborations in the study of inflammatory abatement in acute lung injury. (A) Co‐occurrence map of 129 cocited journals with more than one issue. (B) Co‐occurrence network visualization map of 183 cocited journals with more than 20 citations. (C) Dual map overlay of journals with inflammation subsiding in acute lung injury research.

**Figure:**
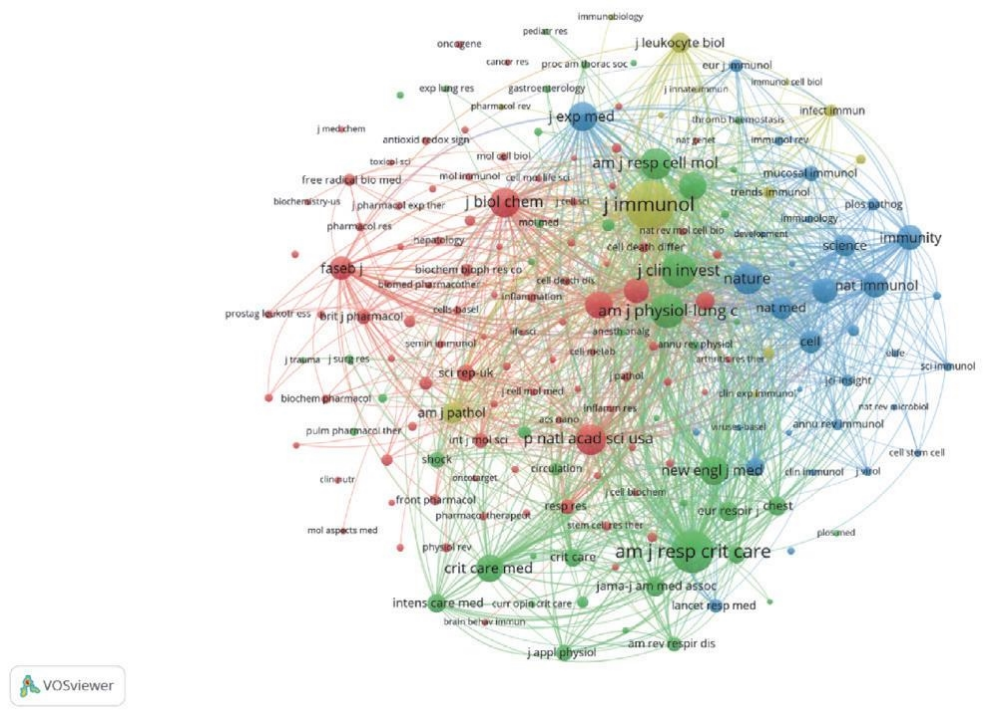
(A)

**Figure:**
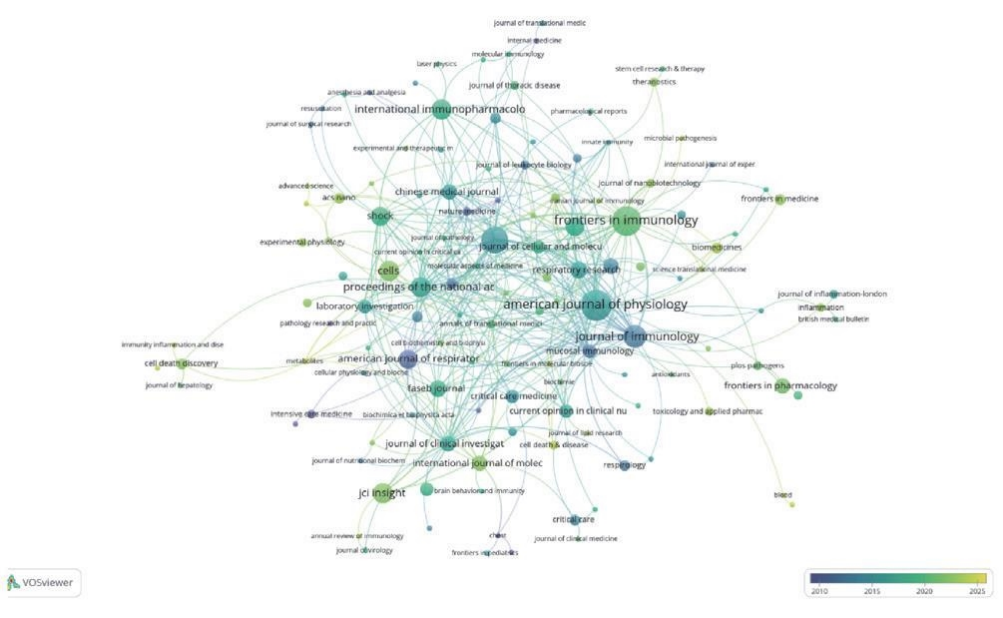
(B)

**Figure:**
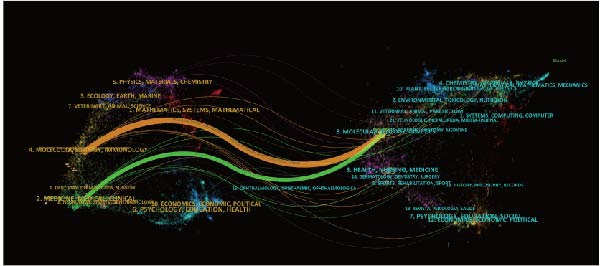
(C)

**Table 4:** Top 10 journals in terms of number of publications.

Rank	Journal	Publications	TC	H‐index	G‐index	IF (2024)
1	*American Journal of Physiology-Lung Cellular and Molecular Physiology*	16	1285	13	16	3.6
2	*Frontiers in Immunology*	15	1329	11	15	5.7
3	*American Journal of Respiratory and Critical Care Medicine*	13	1304	12	13	19.3
4	*Journal of Immunology*	10	948	10	10	3.6
5	*Proceedings of the National Academy of Sciences of The United States of America*	8	864	8	8	9.4
6	*Cells*	8	238	7	8	5.1
7	*JCI Insight*	8	173	6	7	6.3
8	*International Immunopharmacology*	8	105	5	8	4.8
9	*American Journal of Respiratory Cell and Molecular Biology*	7	409	7	7	5.9
10	*Shock*	7	185	6	7	2.7

### 3.5. Outbreak of Cocited References and Reference Citations

Cocited references are references that are cited in more than one publication [26]. Table 5 summarizes 10 frequently cited publications within the field of acute lung injury and inflammation abatement, where Resolvin D1 (RvD1) and RvE1 play a central role in mitigating inflammation as SPMs. Among these literatures, an article entitled “CD4+CD25+Foxp3+ Tregs resolve experimental lung injury in mice and are present in humans with acute lung injury” received the most citations [27]. CiteSpace categorized the references into 20 clusters and included only the top nine clusters: #airway inflammation, #sepsis‐associated lung injury, #regulatory t cell, #skeletal muscle dysfunction, #lung injury, #lung inflammation, #using SPM, #acute pulmonary inflammation, #endothelial cell (Figure 6A). Figure 6B displays a network diagram of references cited in the literature, with each node representing a cited reference and its size reflecting the citation frequency of that reference. Connections between nodes represent cocitation relationships, and the color gradient from purple to yellow denotes different years, spanning from 2005 to 2024. Using CiteSpace with the parameters set to “The Number of States: 2” and “Minimum Duration: 2,” we have identified 24 highly‐cited references and selected the top 20 from them. In Figure 6C, the blue lines represent the timeline, while the red lines indicate the intervals of citation bursts, demonstrating the intensity of these bursts. The first reference with a citation burst (strength = 3.48) appeared in 2006, and the reference that has experienced the most recent citation surge, with a strength of 3.73, was published in the year 2021.

Figure 6Key references. (A) Reference clustering using CiteSpace. (B) A network analysis of cocited references. (C) Highlighting the top 20 references exhibiting the most notable surge in citations.

**Figure:**
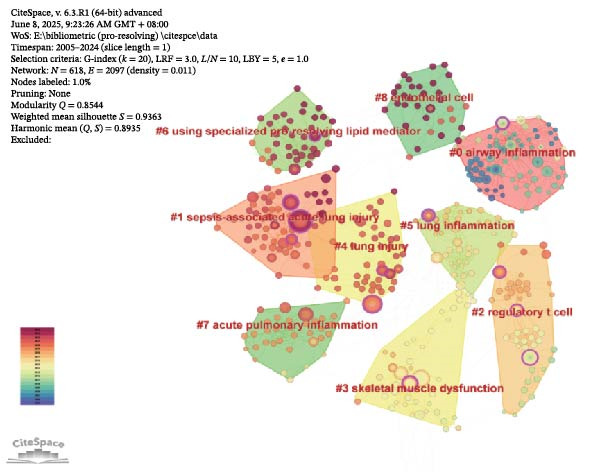
(A)

**Figure:**
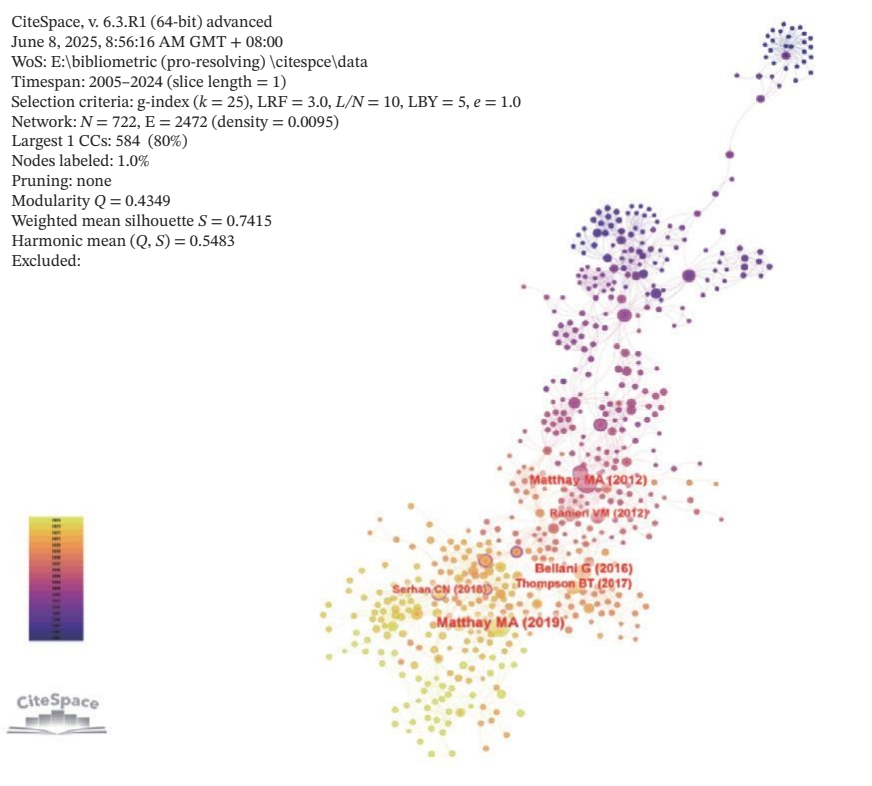
(B)

**Figure:**
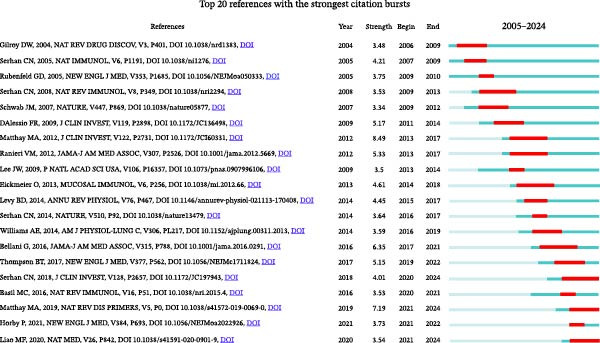
(C)

**Table 5:** Top 10 cited literature.

Rank	Title	Corresponding author	Journal (IF 2024)	Publication year	Total citations (*n*)
1	CD4+CD25+Foxp3+ Tregs resolve experimental lung injury in mice and are present in humans with acute lung injury	D’Alessio FR	*Journal of Clinical Investigation* (IF = 13.3)	2009	27
2	Aspirin‐triggered resolvin D1 reduces mucosal inflammation and promotes resolution in a murine model of acute lung injury	Eickmeier O	*Mucosal Immunology* (IF = 7.9)	2013	23
3	15‐Epi‐lipoxin A4 inhibits myeloperoxidase signaling and enhances resolution of acute lung injury	Driss El Kebir	*American Journal of Respiratory and Critical Care Medicine* (IF = 19.3)	2008	21
4	Diverse macrophage populations mediate acute lung inflammation and resolution	Neil R Aggarwa	*American Journal of Physiology-Lung Cellular and Molecular Physiology* (IF = 3.6)	2014	17
5	Acute lung injury: how macrophages orchestrate resolution of inflammation and tissue repair	Susanne Herold	*Frontiers in Immunology* (IF = 5.7)	2011	16
6	The anti‐inflammatory and proresolving mediator resolvin E1 protects mice from bacterial pneumonia and acute lung injury	Hiroyuki Seki	*Journal of Immunology* (IF = 3.6)	2010	15
7	Resolvin E1 promotes phagocytosis‐induced neutrophil apoptosis and accelerates resolution of pulmonary inflammation	Driss El Kebir	*Proceedings of the National Academy of Sciences* (IF = 9.4)	2012	14
8	A novel anti‐inflammatory and proresolving role for resolvin D1 in acute cigarette smoke‐induced lung inflammation	Hsi‐Min Hsiao	*Plos One* (IF = 2.9)	2013	8
9	A2B adenosine receptor signaling attenuates acute lung injury by enhancing alveolar fluid clearance in mice	Tobias Eckle	*Journal of Clinical Investigation* (IF = 13.3)	2008	7
10	Disruption of Nrf2 impairs the resolution of hyperoxia‐induced acute lung injury and inflammation in mice	Narsa M Reddy	*Journal of Immunology* (IF = 3.6)	2009	7

### 3.6. Keyword Analysis of Publications

**Figure 8:**
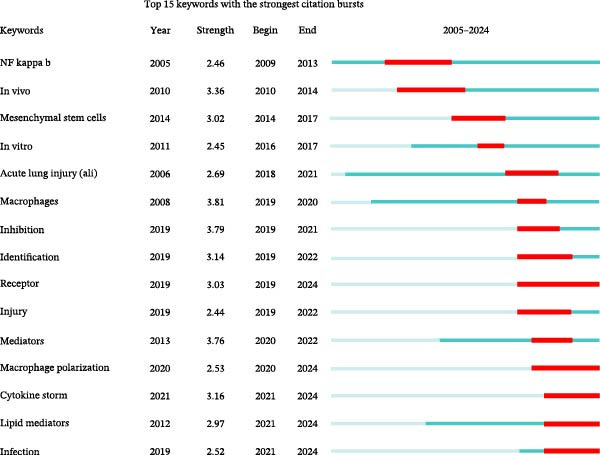
The top 15 keywords exhibiting the most significant surges in citations.

Analyzing the co‐occurrence of keywords is a prevalent technique for exploring trending research areas and disciplines. When conducting analysis in VOSviewer, we consider terms that have been listed as keywords on more than eight occasions, ultimately identifying 87 keywords. These keywords are then clustered into five groups, each represented by a different color in Figure 7A. Cluster 1 and Cluster 2 are primarily composed of terms such as macrophages, regulatory T cells, dendritic cells, and stem cells, which are cells associated with the resolution of inflammation. Cluster 3, Cluster 4, and Cluster 5’s keywords are inflammation, activation, apoptosis, resolution, phagocytosis, express, and lipopolysaccharide (LPS), which are mainly related to the mechanisms of inflammation resolution. In Figure 7B, VOSviewer utilizes the average occurrence time of keywords and represents the timeliness of the keywords with colors. Blue indicates that the keyword emerged earlier, while yellow signifies a more recent appearance. Based on the average publication year (APY), the most recent keywords are “SARS‐CoV‐2” (severe acute respiratory syndrome coronavirus 2) and “COVID‐19” (coronavirus disease 2019). In the field related to mechanisms, the most recent keywords are “lipid mediators” and “macrophage polarization.” The CiteSpace software is capable of clustering related keywords, extracting only the top 10 clusters, and selecting the most representative keywords as cluster names through the log‐likelihood ratio (LLR). In Figure 7C, the recent active clusters in the visualization timeline are #2 lipid mediators, indicating that lipid mediators are likely to be a hot topic in the study of inflammation resolution in lung injury. Figure 8 identifies the top 15 keywords that have experienced the most significant citation surges, with recent spikes in citations including “cytokine storm,” “lipid mediators,” and “infection.”

Figure 7Main keywords. (A) Eighty‐seven keywords co‐occurrence analysis more than eight times. (B) Time visualization of 87 keywords co‐occurrence analysis. (C) Timeline plot of keyword clustering.

**Figure:**
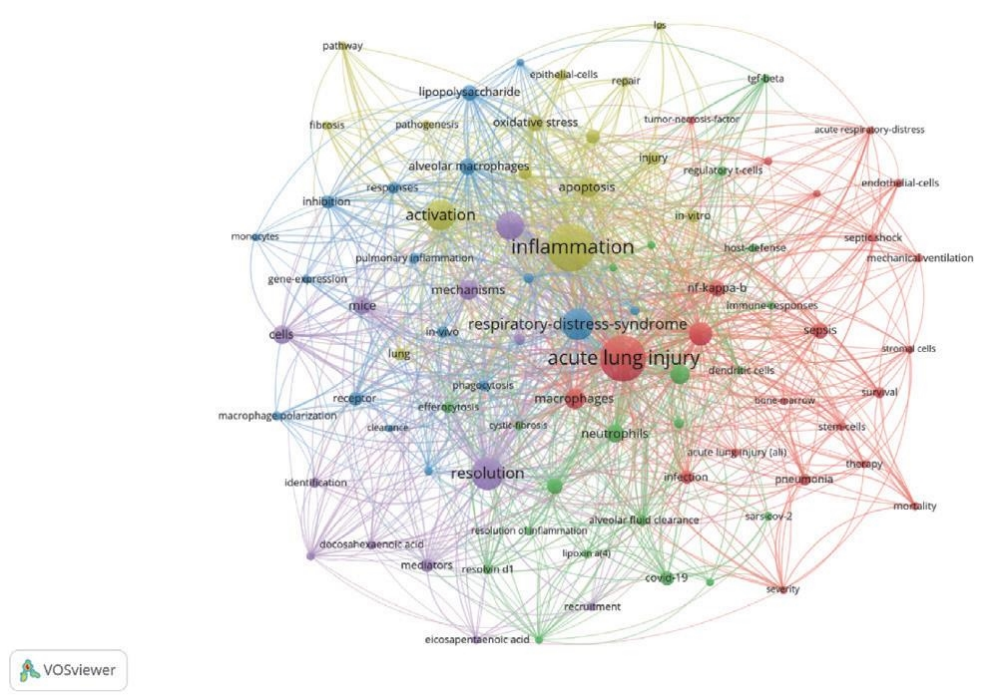
(A)

**Figure:**
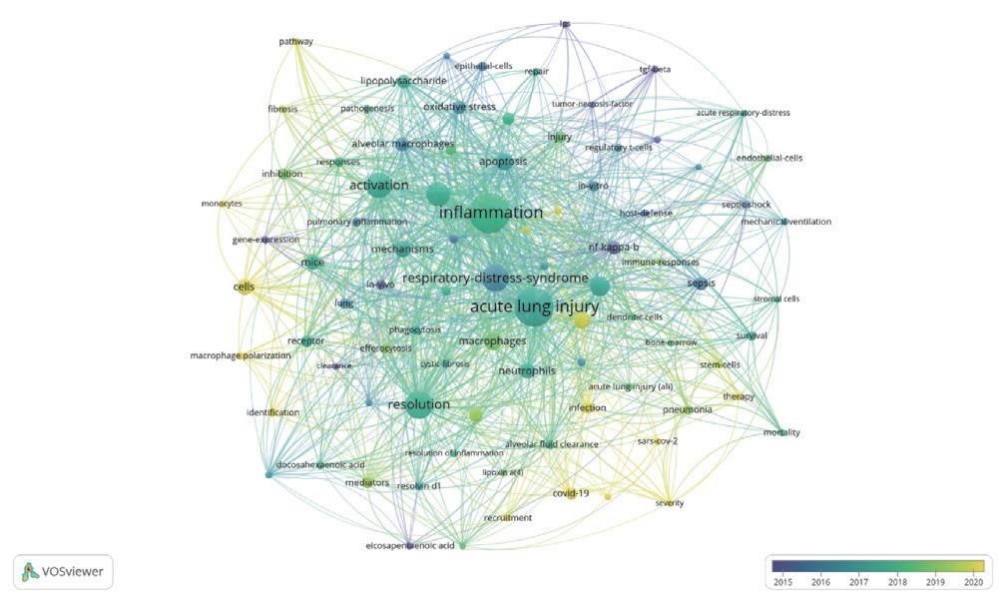
(B)

**Figure:**
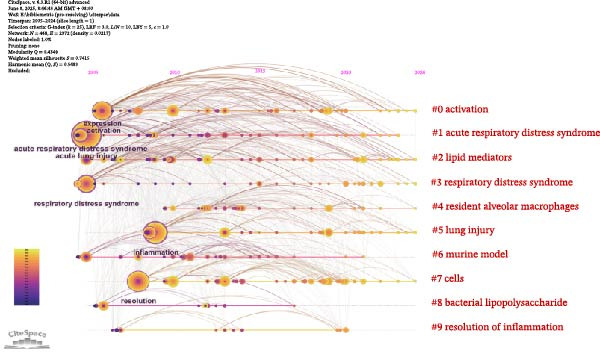
(C)

## 4. Discussion

### 4.1. Overview Details

Within the scope of this research, we utilized bibliometrics to analyze lung injury in the context of inflammation resolution, incorporating a total of 375 documents. Research on lung injury in the context of inflammation resolution has shown a steady upward trend from 2005 to 2024, peaking in the year 2022. A total of 179 countries, 1400 institutions, and 2711 authors have contributed to this field. Chinese scholar JIN SW is a leading researcher in the field of acute lung injury therapy, focusing on the role of endogenous lipid mediators, such as Rvs, PDs, and MaRs, in the regression of inflammation in lung injury [4, 28, 29]. Nationally, the United States and China stand out as the leading contributors, with the United States publishing the highest number of articles. Harvard University has the highest mediated centrality, and among the top 10 global institutions ranked by mediated centrality, seven are situated in the United States. This is one of the key factors that has enabled the USA to become a global leader in research. The American Journal of Physiology‐Lung Cellular and Molecular Physiology leads in terms of publication volume and citation frequency, while the American Journal of Respiratory and Critical Care Medicine claims the top impact factor. This journal enjoys a prestigious academic status and significant influence in the field of respiratory medicine, being one of the widely cited top‐tier journals in the domain. Current research in this field often focuses on molecular biology and genetics, while there is a relative scarcity of related articles in clinically oriented journals. To bridge the gap between basic scientific research and clinical practice, there is an urgent need to promote interdisciplinary collaboration.

### 4.2. Analysis of Research Hotspots and Frontiers

Out of the 10 most frequently cited articles, four concentrate on the study of RvD1 and RvE1, which are bioactive lipids classified under SPMs. These SPMs, including the E‐series derived from eicosapentaenoic acid (EPA), the D‐series derived from docosahexaenoic acid (DHA), and the T‐series derived from docosapentaenoic acid (DPA), are synthesized from essential omega‐3 fatty acids through the action of specific enzymes such as cyclooxygenases and lipoxygenases [30, 31]. In the pathological processes of ALI/ARDS, endogenous lipid mediators may be insufficient to counteract the production of pulmonary inflammation or to prompt a timely resolution of inflammation. Therefore, the intervention of exogenous proresolving lipid mediators may have a protective effect on lung tissue and help to balance the inflammatory response. Previous studies have confirmed that RvD1 exhibits potent anti‐inflammatory and proresolving effects in animal models of multiple ulcerative colitis, acute pancreatitis, and ALI [32–34]. Studies have demonstrated that aspirin‐triggered RvD1 (AT‐RvD1) in a mouse model of acute lung injury significantly reduces the levels of proinflammatory cytokines in bronchoalveolar lavage fluid (BALF), including interleukin (IL)‐1β, IL‐6, Kupffer cells, and tumor necrosis factor‐α, thereby promoting the resolution of inflammation [35]. Additionally, RvD1 enhances the self‐renewal and phagocytic activity of resident alveolar macrophages (RAMs) and facilitates inflammation resolution through the ALX/MAPK14/S100A8/A9 signaling pathway [36]. Meanwhile, RvD1 inhibited MAPKs and NF‐κB pathways through selective responses with ALX receptors, improved survival, and attenuated ALI in LPS‐induced mice [37]. Prof. Driss El Kebir’s study showed that administration of RvE1 at the peak of inflammation promotes neutrophil apoptosis via the caspase pathway and accelerates the process of inflammatory regression in three mouse models of acute lung injury [9]. This suggests that RvE1 contributes to accelerated inflammatory regression by promoting programmed cell death in neutrophils. These findings emphasize the potential value of RvD1 and RvE1 in the treatment of acute lung injury and provide a scientific basis for the development of new therapeutic strategies.

The keyword timeline visualizations in Figure 7B, C, along with the citation burst diagrams in Figure 8, illustrate the evolution of research focuses within the field across different periods. From the early keywords indicated and marked in green in Figure 7B such as “acute lung injury,” “neutrophil,” and “resolvin D1” to the recent keywords marked in yellow such as “SARS‐CoV‐2,” “COVID‐19,” and “lipid mediators,” it can be seen that the focus of research has evolved over time from fundamental pathology and immune responses to the current global health crisis, particularly the COVID‐19 pandemic, as well as the associated inflammatory and immune regulatory factors. In particular, lipid mediators such as GM3‐rich exosomes may play an important role in the pathogenesis of COVID‐19. Studies have indicated that the plasma lipid profile of individuals with COVID‐19 is similar to that of exosomes rich in monosialodihexosyl ganglioside (GM3), characterized by elevated concentrations of sphingomyelins (SMs) and GM3s and reduced levels of diacylglycerols (DAGs) [38]. This suggests that GM3‐enriched exosomes may be involved in metabolic dysregulation associated with disease severity during COVID‐19 pathology.

**Figure 9:**
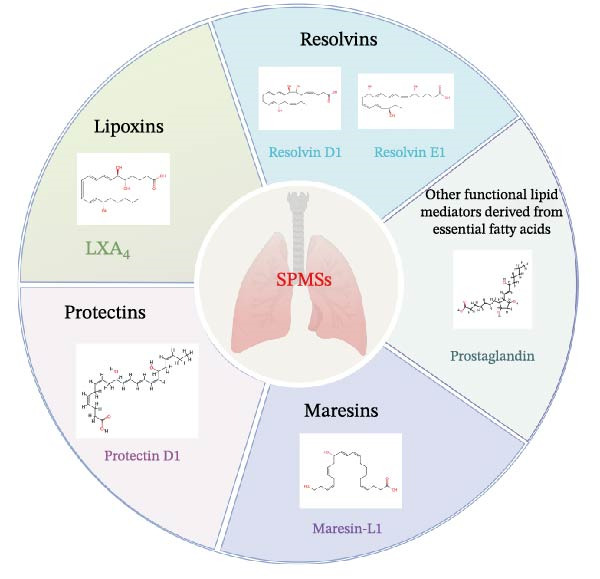
The classification of the SPM family.

In the timeline visualization of Figure 7C, the recently active cluster (#7 lipid mediators) is particularly prominent in lung injury inflammation regression. Meanwhile, in the keyword outbreak map, keywords such as “cytokine storm,” “infection,” and “lipid mediators” remain highly active, further highlighting the importance of lipid mediators in this research field. SPM encompasses four major categories: LXs, Rvs, PDs, and MaRs, in addition to other functional lipid mediators derived from essential fatty acids [39] (Figure 9). These bioactive lipids, primarily originating from omega‐3 polyunsaturated fatty acids, play a crucial role in mitigating inflammatory reactions [40]. The main effects of SPMs include preventing further aggregation of neutrophils, stimulating the release of proinflammatory cytokines, decreasing the levels of proinflammatory cytokines, and increasing the efficiency of clearance of microbial and cellular debris [41]. Lipid mediators such as prostaglandins, leukotrienes, and platelet‐activating factors play a dual role in the inflammatory response, as they are involved in both triggering and intensifying inflammation, as well as in the resolution of inflammatory processes [42]. The regulatory effects of lipid mediators are particularly significant in lung injury models. They help to reduce lung injury, improve lung function, and promote the regression of inflammation by promoting apoptosis of inflammatory cells, regulating migration and activation of immune cells, and promoting tissue repair, among other mechanisms [43–46]. Furthermore, it was shown that SPMs were able to enhance Na, K‐ATPase activity by upregulating the levels of ENaC, Na, K‐ATPase, and water channel proteins in alveolar epithelial cells, which in turn promote alveolar fluid clearance (AFC) in ARDS [47–50]. These findings provide new therapeutic strategies for the reduction of inflammation in ALI/ARDS and may help improve patient outcomes and prognosis.

### 4.3. Application of Drug Delivery Systems (DDSs) in the Resolution of Inflammation in ALI/ARDS

DDSs are a key area of research in modern pharmaceutical science, utilizing multidisciplinary approaches to precisely deliver drugs to target sites, thereby modulating the pharmacokinetics, efficacy, toxicity, immunogenicity, and bioidentification of drugs [51]. In recent years, DDS has made significant progress in enhancing drug efficacy, reducing adverse reactions, and ensuring medication safety. RvD1 and RvE1, as endogenous substances that promote the resolution of inflammation, hold great potential; however, they face challenges such as biological instability and lack of targeting when administered exogenously. To address these challenges, researchers have developed various DDS, such as platelet‐embedded liposomes, which release RvD1 in response to ROS at the site of injury, achieving localized controlled release and integrating drug protection, targeting, and controlled release [52]. The Shanghai Institute of Materia Medica, Chinese Academy of Sciences, in collaboration with Anhui University of Traditional Chinese Medicine, has developed a dry powder inhaler sensitive to reactive oxygen species, which significantly improves the treatment efficiency of ALI/ARDS [53]. Sichuan University’s West China Hospital, in conjunction with Harvard Medical School, has developed a black phosphorus nanosheet (BPNS) DDS, which delivers RvD1 to diseased macrophages through targeted peptide modification, enhancing the efficacy against atherosclerosis and providing new strategies for the treatment of ALI/ARDS and other inflammatory diseases [54]. These studies demonstrate that targeted delivery of RvD1 and RvE1 not only enhances drug efficacy but also reduces side effects, opening up new avenues for the clinical treatment of ALI/ARDS.

### 4.4. Limitations

Firstly, by focusing solely on the WOSCC database, we may have missed relevant studies in other key databases like PubMed, Scopus, and Embase. Each of these databases has unique strengths: PubMed excels in biomedical literature, Scopus covers a broad multidisciplinary range, and Embase is strong in pharmaceutical and biomedical research. Not including them could limit the comprehensiveness and breadth of our findings, potentially omitting valuable data and perspectives. Second, our selection of studies was limited to English‐language publications, which may have excluded important findings in the non‐English‐language literature, resulting in a lack of representativeness of our findings in the multilingual literature. In addition, the cut‐off date for our literature search was a specific point in time, which means that studies published after this date were not included, potentially missing out on the latest findings and trends.

## 5. Conclusion

From 2005 to 2024, a total of 375 research papers on the resolution of inflammation following lung injury were published. These papers not only shed light on the present research landscape but also indicate future research trends. Over these two decades, the number of related papers has continued to grow, with the United States being the main contributor to research. JIN SW is a leading figure in this field, Harvard University holds a pivotal position in this area, and the American Journal of Physiology‐Lung Cellular and Molecular Physiology is the publication that disseminates the highest volume of research in this area. RvD1 and RvE1 are crucial in the resolution of inflammation in ALI/ARDS. Their targeted application through DDSs provides new strategies for clinical treatment, reducing side effects and enhancing therapeutic efficacy.

## Author Contributions

**Zixin Luo and Nan Huang**: conceptualization, methodology, data curation, formal Analysis, software, writing – original draft. **Kaihang Luo**: methodology, data curation, resources, software, investigation, supervision, validation, writing – original draft. **Jianhua Li**: software, visualization, formal analysis, supervision, funding acquisition, writing – review and editing. **Chenxi Wang**: validation, visualization, writing – review and editing. **Kang Zou**: conceptualization, investigation, project administration, funding acquisition, resources, writing – review and editing.

## Funding

This work was supported by a grant from the National Natural Science Foundation of China (Grant 82460023), Ganzhou Science and Technology Bureau, China (Grant 2023LNS36644), and Jiangxi Provincial Administration of Traditional Chinese Medicine, China (Grant 2024B0031).

## Disclosure

All authors have seen this version of the manuscript and approved it for submission.

## Conflicts of Interest

The authors declare no conflicts of interest.

## Data Availability

Data sharing is not applicable to this article as no datasets were generated or analyzed during the current study.
